# U-RISC: An Annotated Ultra-High-Resolution Electron Microscopy Dataset Challenging the Existing Deep Learning Algorithms

**DOI:** 10.3389/fncom.2022.842760

**Published:** 2022-04-11

**Authors:** Ruohua Shi, Wenyao Wang, Zhixuan Li, Liuyuan He, Kaiwen Sheng, Lei Ma, Kai Du, Tingting Jiang, Tiejun Huang

**Affiliations:** ^1^Beijing Academy of Artificial Intelligence, Beijing, China; ^2^National Engineering Research Center of Visual Technology, School of Computer Science, Peking University, Beijing, China; ^3^Institute for Artificial Intelligence, Peking University, Beijing, China

**Keywords:** connectomics, EM dataset, deep learning, automatic cell segmentation, transfer learning

## Abstract

Connectomics is a developing field aiming at reconstructing the connection of the neural system at the nanometer scale. Computer vision technology, especially deep learning methods used in image processing, has promoted connectomic data analysis to a new era. However, the performance of the state-of-the-art (SOTA) methods still falls behind the demand of scientific research. Inspired by the success of ImageNet, we present an annotated ultra-high resolution image segmentation dataset for cell membrane (U-RISC), which is the largest cell membrane-annotated electron microscopy (EM) dataset with a resolution of 2.18 nm/pixel. Multiple iterative annotations ensured the quality of the dataset. Through an open competition, we reveal that the performance of current deep learning methods still has a considerable gap from the human level, different from ISBI 2012, on which the performance of deep learning is closer to the human level. To explore the causes of this discrepancy, we analyze the neural networks with a visualization method, which is an attribution analysis. We find that the U-RISC requires a larger area around a pixel to predict whether the pixel belongs to the cell membrane or not. Finally, we integrate the currently available methods to provide a new benchmark (0.67, 10% higher than the leader of the competition, 0.61) for cell membrane segmentation on the U-RISC and propose some suggestions in developing deep learning algorithms. The U-RISC dataset and the deep learning codes used in this study are publicly available.

## Introduction

Accurate descriptions of neurons and their connections are fundamental to modern neuroscience. By depicting neurons with the help of the Golgi-staining method (Golgi, 1885), Cajal proposed the classic “Neuron Doctrine” more than a century ago (y Cajal, 1888), which opened a new era in modern neuroscience. Nowadays, the development of electron microscopy (EM) has enabled us to further explore the structural details of the neural system at nanometer (nm) scales (Shawn, 2016; Kornfeld and Denk, 2018), opening up a new field called, “Connectomics” that aims to reconstruct every single connection in the neural system. One milestone of Connectomics is the *Caenorhabditis elegans* project (White et al., 1986) which maps all 302 neurons and 7,000 connections in a worm. Recently, a small piece of the human cortex was imaged with a high-speed scanning EM, which maps ~50,000 neurons and 110,000,000 synaptic connections (Shapson-Coe et al., 2021). Connectomic data increase exponentially with a higher resolution of EM and a larger neural tissue volume, even reaching the petabyte (PB) scale (Shapson-Coe et al., 2021). Just as it took almost 15 years to complete the connectome of *C.elegans*, the structural reconstruction for higher-level creatures is becoming more and more daunting with the explosion of connectomic data. Among many bottlenecks, accurate annotation from large amounts of EM images is the first one that has to be solved.

**Figure 1:**
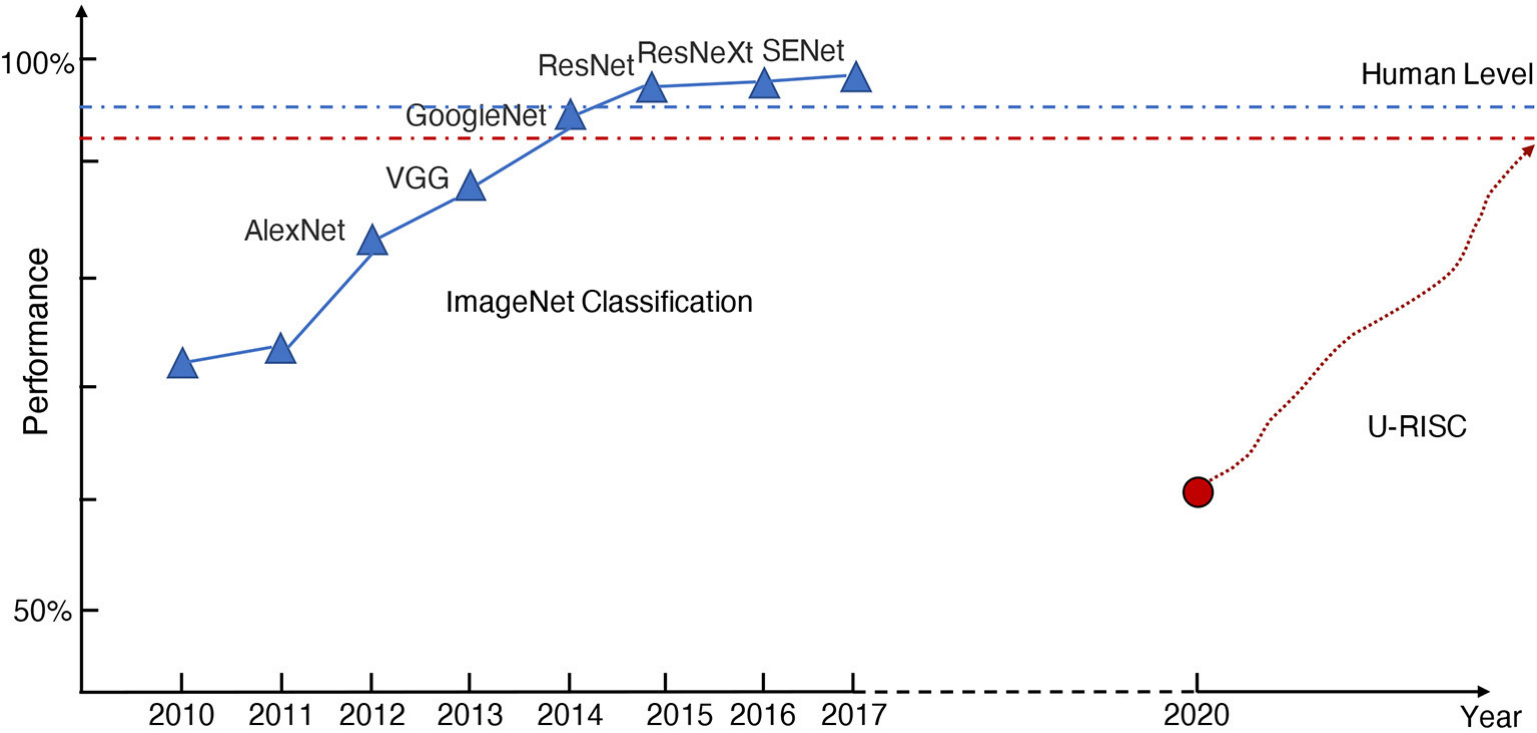
The history of ImageNet. The blue and red dot-dash lines represent the human performance of ImageNet classification and U-RISC segmentation, respectively. The red circle shows the current benchmark performance of U-RISC segmentation, and the red dot line shows the expected improvement of deep learning methods.

Manual annotation of all the connectomic data is infeasible because of the high annotation cost. To reduce the burden of manual annotation for humans, one would hope to enable a machine to annotate the connectomic data with near-human performance automatically. Hopes are higher today because of the rapid development of deep learning methods. However, even with deep learning, it still requires tremendous efforts to achieve human-level performance on this challenging task. There were a few successful experiences to learn from the computer science community to make the deep learning method fully comparable to humans in Connectomics. The success of deep learning methods highly depends on the amount of training data and the quality of annotation. For example, in the task of image classification, ImageNet (Russakovsky et al., 2015) has set up a research paradigm in applying deep learning methods for vision tasks. In 2009, by releasing a large-scale accurately annotated dataset, ImageNet provided a benchmark (72%) for image classification. From 2010 to 2017, a challenge called, “The ImageNet Large Scale Visual Recognition Challenge (ILSVRC)” was organized every year. This challenge significantly boosted the development of deep learning algorithms. Many champions of this challenge have become the milestones for deep learning methods, such as AlexNet (Krizhevsky et al., 2012), VGG (Simonyan and Zisserman, 2014), GoogleNet (Szegedy et al., 2015), and ResNet (He et al., 2016). As shown in Figure 1, deep learning performance on image classification finally exceeded the human level (95%) after 8 years of development. To summarize, there is a roadmap for the success of ImageNet, which includes three key steps: the first step is to establish a large-scale dataset with high-quality annotation, which is very important for deep learning. Based on the dataset, the second step organizes a challenge that can evaluate algorithms at a large scale and allow researchers to estimate the progress of their algorithms, taking advantage of the expensive annotation effort. The third step is the design of new algorithms based on the previous two steps. Each of the three stages is indispensable.

Following the success of ImageNet, significant progress in the automatic segmentation of EM was achieved by the 2012 IEEE International Symposium on Biomedical Imaging (ISBI 2012), which was the first challenge on the automatic segmentation of EM in releasing a publicly available dataset (Arganda-Carreras et al., 2015). The state-of-the-art (SOTA) methods exhibited an unprecedented accuracy in EM cellular segmentation on the dataset of ISBI 2012. In particular, the deep learning method, “U-Net,” (Ronneberger et al., 2015) which was first proposed during the challenge, becomes the backbone of many SOTA methods in the field. However, today many deep learning methods have become “exceedingly accurate,” and are likely to be saturated at the ISBI 2012 (Arganda-Carreras et al., 2015). In addition, ISBI 2012 images are 512 × 512 pixels with a resolution of 4 × 4 nm/pixels, while there are many EM images with higher resolution in connectomics because enough high resolution is essential to unravel the neural structures unambiguously. For instance, 2 nm has been suggested as the historical “gold standard” to identify synapses (DeBello et al., 2014), in particular, to identify gap junctions (Leitch, 1992), which are common in the neural tissues (Anderson et al., 2009). It is not clear if previous classic deep learning methods developed on the EM images with relatively lower resolution can still work well on datasets with higher resolutions.

Here, to promote the deep learning algorithms in EM datasets, we initiated a new roadmap: We first annotated the retinal connectomic data, RC1, from rabbit (Anderson et al., 2011) and presented a brand new annotated EM dataset named, ultra-high resolution image segmentation dataset for cell membrane (U-RISC). Compared to ISBI 2012, the U-RISC has a higher resolution of 2.18 nm/pixel and a larger size of 9,958 × 9,959 pixels. The precision of the annotation was ensured by multi-steps of iterative verification, costing over 10,000 labor hours in total. Next, based on the U-RISC, a competition of cellular membrane prediction was also organized. Surprisingly, from 448 domestic participants/teams, it was observed that the top performance of deep learning methods on the U-RISC (~0.6, F1-score) was far below the human-level accuracy (>0.9), in contrast to the near-human performance of deep learning methods in ISBI 2012. We then made fair comparisons between ISBI 2012 and U-RISC with the same segmentation methods, including U-Net. The comparison results confirmed that U-RISC indeed provides new challenges to the existing deep learning methods. The U-Net, for example, dropped from 0.97 in ISBI 2012 to 0.57 in the U-RISC. To further explore how these methods work on segmentation tasks, we introduced a gradient-based attribution method, an integrated gradient (IG; Sundararajan et al., 2017), to analyze ISBI 2012 and the U-RISC. The result showed that when deciding on whether a pixel belonged to a cell membrane or not, deep learning methods represented by the U-Net would refer to a larger attribution region on the U-RISC (about four times on average) than that on ISBI 2012. This suggests that the deep learning methods might require more background information to decide the segmentation of the U-RISC dataset. Finally, we integrated the currently available advanced methods, combining the U-Net and transferring the learning recently introduced (Conrad and Narayan, 2021), and provided a benchmark (0.6659), which is about 10% higher than the leader board (0.6070), for the U-RISC.

Overall, our contribution in this study lies mainly in the following three parts: (1) we provided the community with a brand new publicly available annotated mammalian EM dataset with the highest known resolution (~2.18 nm/pixel) and the largest image size (9,958 × 9,959 pixels); (2) we organized a competition and made a comprehensive analysis to reveal the challenges of U-RISC in the deep learning methods; (3) we improved the benchmark with 10% to the F1-score of 0.6659. In the Discussion, we proposed further suggestions for improving the segmentation methods from the perspectives of model design, loss function design, data processing, etc. We hope our dataset and analysis can help researchers gain insights into designing more robust methods, which can finally accelerate the speed of untangling brain connectivity.

## Materials and Methods

### Datasets

The U-RISC dataset was annotated upon RC1, a large-scale retinal serial section transmission electron microscopic (ssTEM) dataset, publicly available upon request and described in detail in the study of Anderson et al. (2011). The RC1 came from the retina of a light-adapted female Dutch Belted rabbit after *in vivo* excitation mapping. The imaged volume represents the retinal tissue with a diameter of 0.25 mm, spanning the inner nuclear, inner plexiform, and ganglion cell layers. Serial EM sections were cut at 70–90 nm with a Leica UC6 ultramicrotome and captured at the resolution of 2.18 nm/pixel across both axes using SerialEM (Mastronarde, 2005). In RC1, there are in total 341 EM mosaics generated by the NCR Toolset (Anderson et al., 2009), and we clipped out 120 images in the size of 9,958 × 9,959 pixels from the randomly chosen sections.

To annotate cell membrane with high quality on the 120 images, we launched an iterative annotation project that lasted for 3 months. All the annotators were trained to recognize and annotate cellular membrane in EM images, but only two-thirds of all, 53 annotators, were finally qualified to participate in the project according to their annotation results. In the iterative annotation procedure, each EM image had undergone three continuous rounds of annotation with the guidance of blind review. The final round of annotation was regarded as the “ground truth.” While the first two rounds are valuable for analyzing the human learning process, we also reserved the intermediate results for public release. All of the U-RISC datasets are released at https://github.com/EmmaSRH/U-RISC-Data-Code.

### Competition

The goal of the competition was to predict cell membranes in the EM images of U-RISC. Participants were required to return images depicting the boundary of all neurons. F1-score was selected as the evaluation criterion for the accuracy of the results (Formula 1) (Sasaki and Fellow, 2007). During the evaluation processing, according to the classes of prediction and ground truth, the predicted pixels of images were first divided into four types: true positive (TP), true negative (TN), false positive (FP), and false negative (FN). Then, two metrics, precision and recall, were calculated from the number of these types of pixels. The F1-score was defined as the harmonic mean of precision and recall.


(1) $$\begin{array}{l} \text{F}1-\text{score }=\frac{2\times \text{Precision}\times \text{Recall}}{\text{Precision}+\text{Recall}}. \end{array}$$

(2) $$\begin{array}{l} \text{Precision }=\frac{\text{TP}}{\text{TP}+\text{FP}},\text{ Recall }=\;\frac{\text{TP}}{\text{TP}+\text{FN}}. \end{array}$$

There were two tracks in the competition; images in Track 1 were kept in their original size (9,958 × 9,959 pixels), images in Track 2 were downsampled to the size of 1,024 × 1,024 pixels. Fifty images, 30 as the training dataset and 20 as the test dataset, were released in Track 1. Additionally, Track 2 contained 70 images in total, amounting to 40 training images and 30 testing images. The training dataset included EM images with their corresponding ground truth, while the ground truth of the test dataset was kept private. In both the tracks, ten images from the training dataset served as the validation dataset for the participants to monitor and develop their models. No statistical methods were used to determine the assignment of images in the whole arrangement.

### Segmentation Networks

We conducted experiments to compare the performance of the same methods on U-RISC (Track 2) and ISBI 2012. Three representative deep learning networks such as (**Table 2**), U-Net (Ronneberger et al., 2015), LinkNet (Chaurasia and Culurciello, 2017), and CASENet (Yu et al., 2017) were considered. The three networks are all pixel-based segmentation networks. Specifically, given the input image *x*, the goal of the networks is to classify the corresponding semantic cell membrane pixel by pixel. For the input image *x* and the classification function *F*(*x*), *Y*{*p*|*X*, Θ}, Θ ∈ [0, 1] is taken as the output of the network, which represents the edge probability of the semantic category of the pixel *p*. Θ are the parameters in the network and are optimized in the training process. Architectures of the three networks are described as follows.

#### U-Net

The U-Net (Ronneberger et al., 2015) is a classical fully convolutional network (i.e., there is no fully connected operation in the network). The model is composed of two parts: contracting path and expansive path. The contracting path follows the typical architecture of a convolutional network. At each downsampling step, the U-Net doubles the number of feature channels to gain a concatenation with the correspondingly cropped feature map from the contracting path. At the final layer, a 1 × 1 convolution is used to map each 64-component feature vector to the desired number of classes. In total, the network has 23 convolutional layers. We use ResNet50 as its encoder.

#### LinkNet

The model structure of LinkNet (Chaurasia and Culurciello, 2017) is almost similar to the U-Net, which is a typical encoder–decoder structure. The encoder starts with an initial block which performs convolution on the input image with a kernel of size 7 × 7 and a stride of 2. This block also performs spatial max-pooling in an area of 3 × 3 with a stride of 2. The later portion of the encoder consists of residual blocks and is represented as the encoder-block. To reduce parameters, the LinkNet uses ResNet18 as its encoder.

#### CASENet

**The** CASENet (Yu et al., 2017) is an end-to-end deep semantic edge learning architecture adopting ResNet-152 as its backbone. The classification module here consists of a 1 × 1 convolution and a bilinear interpolation upsampling layer to generate M active images; each image size is the same as the original image. Each residual block is followed by a classification module to obtain five classification activation graphs. Then, a sliced concatenation layer is used to fuse the M classification activation graphs, and finally, a 5M-channel activation graph is obtained. The activation graphs are used as the input for the fused classification layer to obtain an M-channel activation graph. The fusion classification layer is the convolution of the M group, 1 × 1.

#### Transfer Learning

The pretrained model from Conrad and Narayan (2021) was used in our method, specifically, MoCoV2 (Arar et al., 2020) and CEM500K (Conrad and Narayan, 2021) were respectively selected as the pretraining method and dataset.

#### Training Settings

For each dataset, the same training and testing data distribution was utilized for the three methods. For U-RISC, during the training, the original images were cut into 1,024 × 1,024 patches with overlaps. Additionally, the patches were randomly assigned to the training set and validation set according to the ratio of 50,000/20,000. For ISBI 2012, 20 images were used for training, and 10 images were used for testing.

#### Loss Function and Optimization

The U-RISC image membrane segmentation task can be defined as the pixel-level classification task. The ground truth of each pixel is a binary value *y* ∈ {0, 1}, and *y*′ is the predicted value by the prediction model. *Y* is the set of all pixels of one image. For each algorithm, we used the same loss function and optimization method. Specifically, focal loss and dice loss were chosen. Focal loss and dice loss are defined as:


(3) $$\begin{array}{l} L_{\text{Focal}}=\underset{Y}{\sum }-{\left(1-y^{\prime }\right)}^{\gamma }\log\left(y^{\prime }\right). \end{array}$$

(4) $$\begin{array}{l} L_{\text{Dice}}=\underset{Y}{\sum }\frac{2y^{\prime }y+1}{y^{\prime }+y+1}. \end{array}$$

The final loss function is the summation of the two losses with the proportion of 1: λ. That is *L* = *L*_*Focal*_ + λ*L*_*Dice*_. We set λ = 1 and γ = 2 in our experiments. When optimizing the parameters in the network, we chose Adam (Kingma and Ba, 2014) as the optimizer.

#### Implementation Details

Data augmentation (random horizontal/vertical flip, random rotation, random zoom, random cropping, random cropping, random translation, random contrast, and random color jitter) was used. Four Nvidia V100 GPUs were used for training. In the testing stage, the original images were cut into the same size as the training images, and the patches were tested. These patches were eventually mosaiced back to the original size for evaluation. The parameter settings are shown in Table 1. Mean value and standard error are computed by testing the images of each dataset. The methods with “-^*^” in the table represent that they are implemented by us.

**Table 1:** Implementation details.

**Implementation**	U-Net-^*^	CASENet-^*^	LinkNet-^*^	**U-Net-transfer**
Data augmentation	√	√	√	√
Pre-training	–	–	–	√
Learning rate	1e-3	1e-7	5e-4	2e-5
Batch size	4	2	1	4
GPUs	4	4	4	8
Epoch	100	100	300	50
Worker	16	16	8	32

### Image Definition Criteria

As the competition includes two tracks and the participants have obvious different performances on them, we introduced the four representative image definition criteria, Brenner (Subbarao and Tyan, 1998), SMD2 (Thakkinstian et al., 2005), Variance (Saltelli et al., 2010), and Vollath (2008) to analyze the effects of downsampling on EM images (in discussion and Appendix Figure). The former two consider the difference and variance of gray values between adjacent pixels, while the latter two consider the whole image.

Brenner gradient function simply calculates the square of the gray difference between two adjacent pixels.


(5) $$\begin{array}{l} D\left(f\right)=\underset{y}{\sum }\underset{x}{\sum }|f\left(x+2,y\right)-f\left(x,y\right){|}^{2}. \end{array}$$

where, *f*(*x, y*) represents the gray value of pixel (*x, y*) corresponding to image *f*, and *D*(*f*) is the result of image definition calculation (the same below).

The SMD2 multiplies two gray variances in each pixel field and then accumulates them one by one.


(6) $$\begin{array}{l} D\left(f\right)=\underset{y}{\sum }\underset{x}{\sum }|f\left(x,y\right)-f\left(x+1,y\right)||f\left(x,y\right)-f\left(x,y+1\right)|. \end{array}$$

The variance function is defined as


(7) $$\begin{array}{l} D\left(f\right)=\underset{y}{\sum }\underset{x}{\sum }{\left|f(x,y)-\mu )\right|}^{2}. \end{array}$$

where μ is the average gray value of the whole image, which is sensitive to noise. The purer the image, the smaller is the function value.

The Vollrath function is defined as follows:


(8) $$\begin{array}{l} D\left(f\right)=\underset{y}{\sum }\underset{x}{\sum }f\left(x,y\right)f\left(x+1,y\right)-MN\mu^{2}. \end{array}$$

where μ is the average gray value of the whole image, *M* and *N* are the width and height of the image, respectively.

### Attribution Analysis

We also noticed the different performance of U-Net when applied on ISBI 2012 and U-RISC. To explore the deeper reason, we carry out an attribution analysis on the U-Net by using the IG (Sundararajan et al., 2017) method to quantify the contribution maps (in section Attribution Analysis of the Deep Learning Method on U-RISC and ISBI 2012). For a given input image *x* and model *F*(*x*), the goal of the network is to find out which pixels or features in *x* have an important influence on the decision-making of the model or sort the importance of each pixel or feature in *x*. Such a process is defined as attribution. The IG uses the integrated value along the whole gradient line from the input to the output. In the cell membrane segmentation task, from the decision of a pixel of *y* (predicted as the cell membrane or not), we can obtain the contribution of each pixel of the input image. Putting the contribution of each pixel together, we record it as an attribution field *A*, whose size is the same as the original image. The value *x*_*i*_ denotes the *i*_*th*_ pixel in image *x*, and *w*_*i*_ denotes the attribution value of *x*_*i*_, representing the contribution decision of pixel *x*_*i*_ to *y*. The value of *w*_*i*_ is normalized to [−1,1].

In the binary segmentation task, for the current input image *x*, if we know that the output *y* is a specific value, such as *y* = 0, and the corresponding reference image is *x*′, then we can take a linear interpolation, i.e.,


(9) $$\begin{array}{l} x^{\prime }+\alpha \left(x-x^{\prime }\right). \end{array}$$

If the constant α = 0, then the input image is the base image as that of *x*′. If α = 1, then the input image is the current image, which is *x*. When 0 < α < 1, it can be other images.

For the output of the neural network *F*(*x*), the attribution value of *x*_*i*_, *w*_*i*_ is computed as follows.


(10) $$\begin{array}{l} w_{i}=\left(x_{i}-{x_{i}}^{\prime }\right)\times \int_{\alpha =0}^{1}\frac{\partial F\left(x^{\prime }+\alpha \left(x-x^{\prime }\right)\right)}{\partial x_{i}}d\alpha . \end{array}$$

Here, $\frac{\partial F\left(x\right)}{\partial x_{i}}\;$is the gradient of *F*(*x*) with respect to *x*_*i*_.

As the resolution and image size of U-RISC and ISBI 2012 are different, for a fair comparison, we define the size of the pixel attribution field as *S*_*k*_, which represents the physical size corresponding to the pixel area with the fixed contribution value threshold, *k*. If the attribution value *w*_*i*_ is greater than *k*, the pixel is the one with a higher contribution in decision-making. The area of the attribution field *S*_*k*_ is obtained by multiplying the number of pixels with the attribution value, *w*_*i*_ which is larger than *k* and the corresponding physical size of the pixel (square of resolution *h*).


(11) $$\begin{array}{l} S_{k}=|A_{w_{i}>k}|\times h^{2},\;w_{i}\in A \end{array}$$

### Data Analysis

All statistical tests used, including statistic values and sample sizes, are provided in the figure captions, including the mean and standard. All analyses were performed using custom software developed using the following tools and software: MATLAB (R2018a), Python (3.6), PyTorch (1.6.0), NumPy (1.19.0), SciPy (1.5.1), and matplotlib (2.2.3).

## Results

### The Largest Ultra-High-Resolution EM Cell Membrane Segmentation Dataset

**Figure 2:**
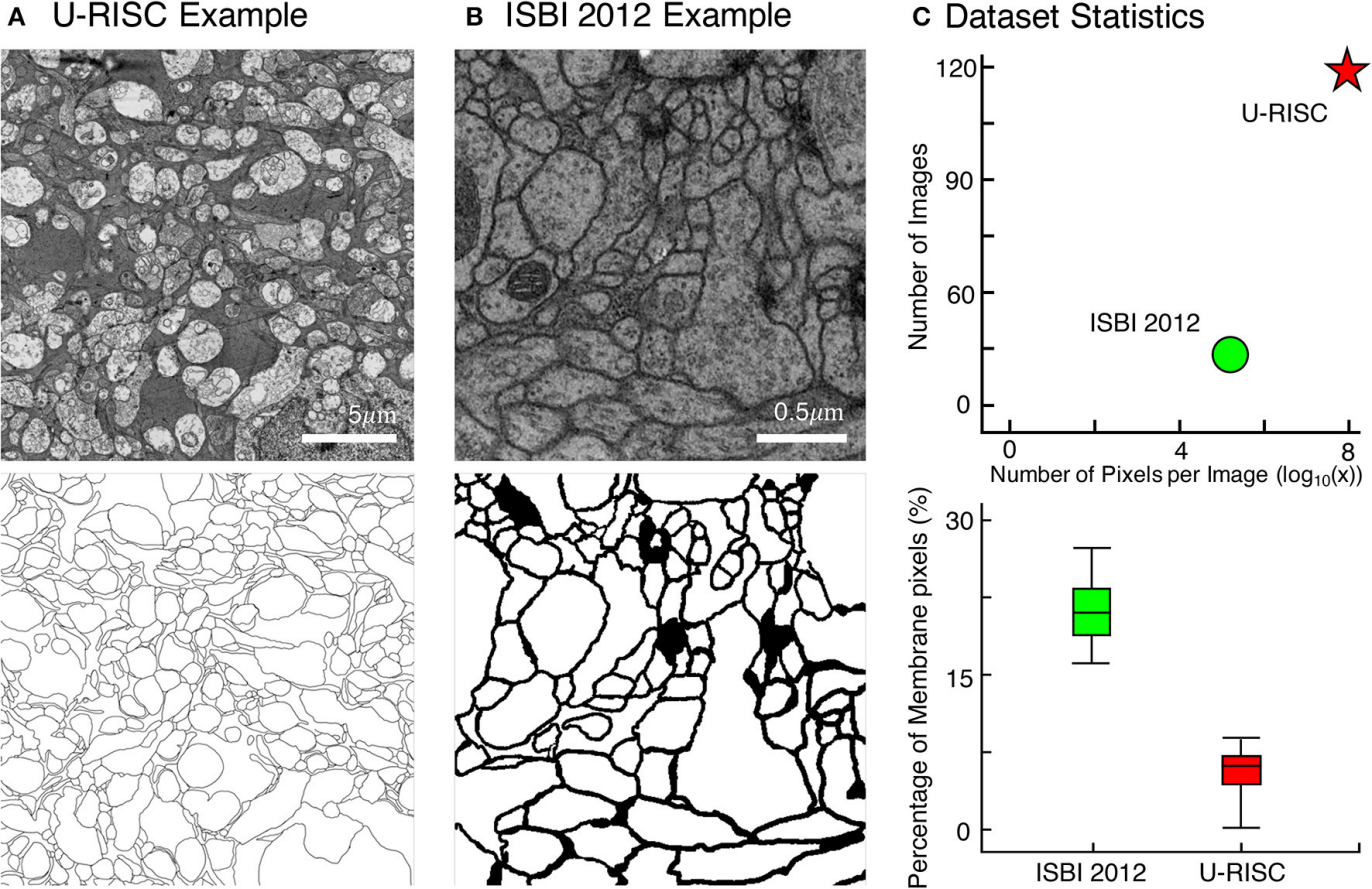
Comparison between U-RISC and ISBI 2012. **(A,B)** An example of U-RISC and ISBI 2012 data includes the raw EM image (top) and the corresponding annotation result (bottom). Black pixels in annotation results represent cellular membranes. **(C)** (Top) Both the number and size of images in U-RISC surpass those in ISBI 2012. (Bottom) The proportion of annotated pixels, 21.65 ± 2% in ISBI 2012 and 5.10 ± 2% in U-RISC, making the latter a more imbalanced dataset.

Along with this article, we proposed a new EM dataset with cell membrane annotated the U-RISC. To our best knowledge, U-RISC has the highest resolution among the publicly available annotated EM datasets (refer to Figure 2A as an example). It was annotated upon the rabbit retinal connectomic dataset RC1 (Anderson et al., 2011) with a 2.18 nm/pixel resolution at both the *x* and *y* axes. Taking ISBI 2012 as an example (Figure 2B) (120 pairs of 9,958 × 9,959 pixel images in the U-RISC and 30 pairs of 512 × 512 pixel images in ISBI 2012) (Figure 2C). One characteristic of U-RISC is that cell membranes only cover a small area of the images, making it an imbalanced dataset for deep learning (an average of 5.10% ± 2% in U-RISC compared to 21.65% ± 2% in ISBI 2012).

**Figure 3:**
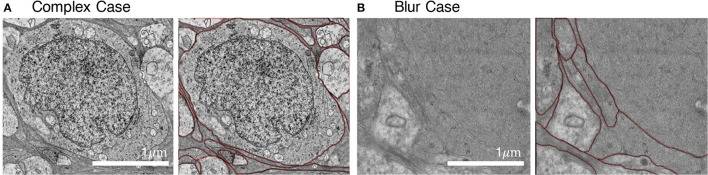
Examples of images with their annotations. **(A)** Organelle membranes were cautiously avoided to be annotated. **(B)** More time and patience were needed to annotate the image with low contrast.

**Figure 4:**
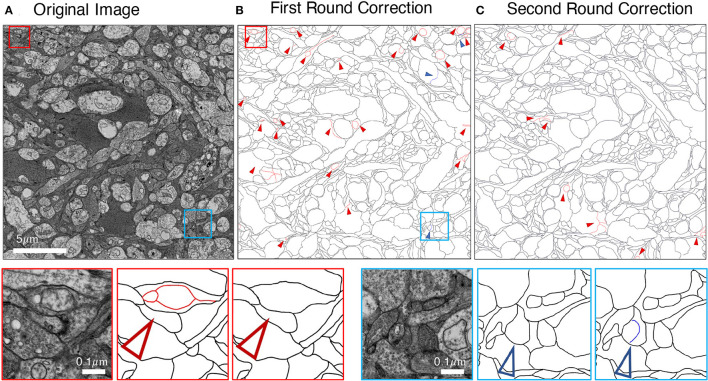
Example of iterative human annotation. **(A)** Original image to be annotated. **(B)** Many errors were found in the first round of annotation. **(C)** After correction, much fewer errors were detected in the second round of annotation, and the correction results were served as the final annotation. Red small triangles and boxes indicate false-positive errors (enlargement in the bottom left), blue for false-negative errors (enlargement in the bottom right).

We employed an iterative manual annotation procedure to ensure the quality of annotation. Because of the difficulty in distinguishing the cell membrane from the organelle membrane, special attention was paid to exclude the organelle membrane from annotation (Figure 3A). In practical connectomic research, the image quality can be affected by many reasons, such as insufficient staining and thick section. Considering this, we retained several images with low quality in the U-RISC to make the dataset closer to the actual situation. Annotation on these images costs more time and caution (Figure 3B). Labeling errors could be detected and then corrected in each round of iteration (Figure 4). For scientific research reasons, the human labeling process is very valuable for uncovering the human learning process. Therefore, the intermediate annotated results were also reserved for public release (https://github.com/EmmaSRH/U-RISC-Data-Code).

### Ultra-High Resolution EM Images Segmentation Competition

To investigate the performance of the deep learning methods on the U-RISC and to propose a benchmark, a competition on cellular membrane segmentation was organized by the Beijing Academy of Artificial Intelligence Institution, Beijing, China (BAAI) and the Peking University, Beijing, China (PKU)^1^. In total, 448 participants took part in the competition, mainly from domestic competitive universities, research organizations, and top IT institutions.

There were two tracks in the competition (Table 2): Track 1 used the original images with the size of 9,958 × 9,959 pixels as training and testing datasets, respectively. In Track 2, the images were downsampled to the size of 1,024 × 1,024 pixels. The purpose of Track 2 was to allow researchers with limited computational resources to participate in the competition. The final round of human annotation was used as the ground truth to evaluate the algorithms, and an F1-score was applied as the evaluation metric (for details, please refer to Methods and Materials).

**Table 2:** Leaderboard of track 1 and track 2.

**Track 1 (original)**			**Track 2 (downsample)**		
**Team name**	**Institution**	**F1-score**	**Team name**	**Institution**	**F1-score**
Human 1st	–	0.92128 ± 0.012	Human 1st	–	0.96915 ± 0.014
Human 2nd	–	0.92128 ± 0.012	Human 2nd	–	0.99891 ± 0.003
SCP173	Tencent^a^	0.60704 ± 0.043	Horch	UCAS^b^	0.56932 ± 0.053
yangsenwxy	SCU^c^	0.60701 ± 0.042	Deadline	NJU^d^	0.56213 ± 0.055
SpongeBobbb	HDU^e^	0.60480 ± 0.042	SpongeBobbb	HDU^e^	0.56136 ± 0.049
VIDAR	USTC^f^	0.60303 ± 0.041	VIDAR	USTC^f^	0.55170 ± 0.046
Deadline	NJU^d5^	0.60066 ± 0.045	Archer	THU^g^	0.55107 ± 0.047
Chasingstar	JLU^h^	0.59647 ± 0.044	scu_ws	SCU^c^	0.54847 ± 0.053

a *Tencent Holdings Ltd (China)*.

b *University of Chinese Academy of Sciences (China)*.

c *Sichuan University (China)*.

d *Nanjing University (China)*.

e *Hangzhou Dianzi University (China)*.

f *University of Science and Technology of China*.

g *Tsinghua University (China)*.

h *Jilin University (China)*.

**Figure 5:**
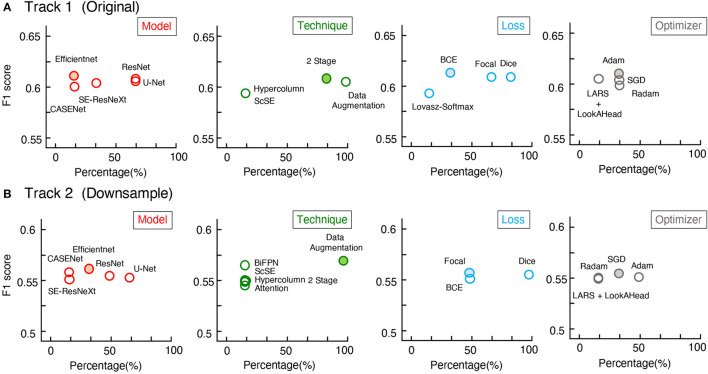
Mean F1-scores of teams with different methods used. **(A,B)** The statistics of Track 1 and Track 2, respectively. The x-axis represents the proportion of the team with the method, the y-axis represents the average of F1-scores.

Surprisingly, from the competition, the top 6 teams in each track gained F1-scores around 0.6 on U-RISC, which were far below the human levels (0.92 and 0.99, the first and second rounds of annotation). However, a previous study has shown that the performance of the top teams in ISBI 2012 had already been reasonably closer to the human level (Arganda-Carreras et al., 2015). To investigate the causes of the performance gap between the methods and humans on the U-RISC, we first surveyed the top 6 teams in our competition. It indicated that a variety of current popular approaches to segmentation were utilized (Figure 5). From the choice of models (Figure 5A), the participants used the current popular image segmentation networks, such as U-Net (Ronneberger et al., 2015), Efficientnet (Tan and Le, 2019), and CASENet (Yu et al., 2017). For backbone selection, the ResNet (He et al., 2016) and their variants were the most chosen architectures. Data augmentation was ubiquitously applied to improve the generalization of the models. About 13% of the participants used Hypercolumns (Hariharan et al., 2015) to improve the expressiveness of the model. From the design of the loss function, functions that can adjust penalty ratios according to sample distributions were applied to reduce the effect of sample imbalance, such as dice loss (Dice, 1945), focal loss (Lin et al., 2017), and BCE loss (Cui et al., 2019). Additionally, Adam (Kingma and Ba, 2014) was shown to be the most chosen optimization method.

The analysis suggested that even though participants had considered many popular methods, their performance was still not satisfactory and varied only slightly between each other. To identify whether this was because of the challenges of U-RISC or the methods themselves, we picked out the three widely used methods, the U-Net (Ronneberger et al., 2015), LinkNet (Chaurasia and Culurciello, 2017), and CASENet (Yu et al., 2017). We conducted a fair comparison between the performance of each method on U-RISC (Track 1) and ISBI 2012. Results showed that these methods could reach over 0.97 (F1-score) in ISBI 2012, but only between 0.57 and 0.61 in the U-RISC (Table 3), which confirmed that the performance gap in competition comes from the challenges of U-RISC.

**Table 3:** F1-scores in U-RISC and ISBI 2012.

**Method**	**U-RISC**	**ISBI 2012**
LinkNet-^*^	0.60701 ± 0.063	0.97246 ± 0.08
CASENet-^*^	0.60065 ± 0.053	0.97132 ± 0.08
U-Net-^*^	0.57123 ± 0.049	0.97010 ± 0.09

**Figure 6:**
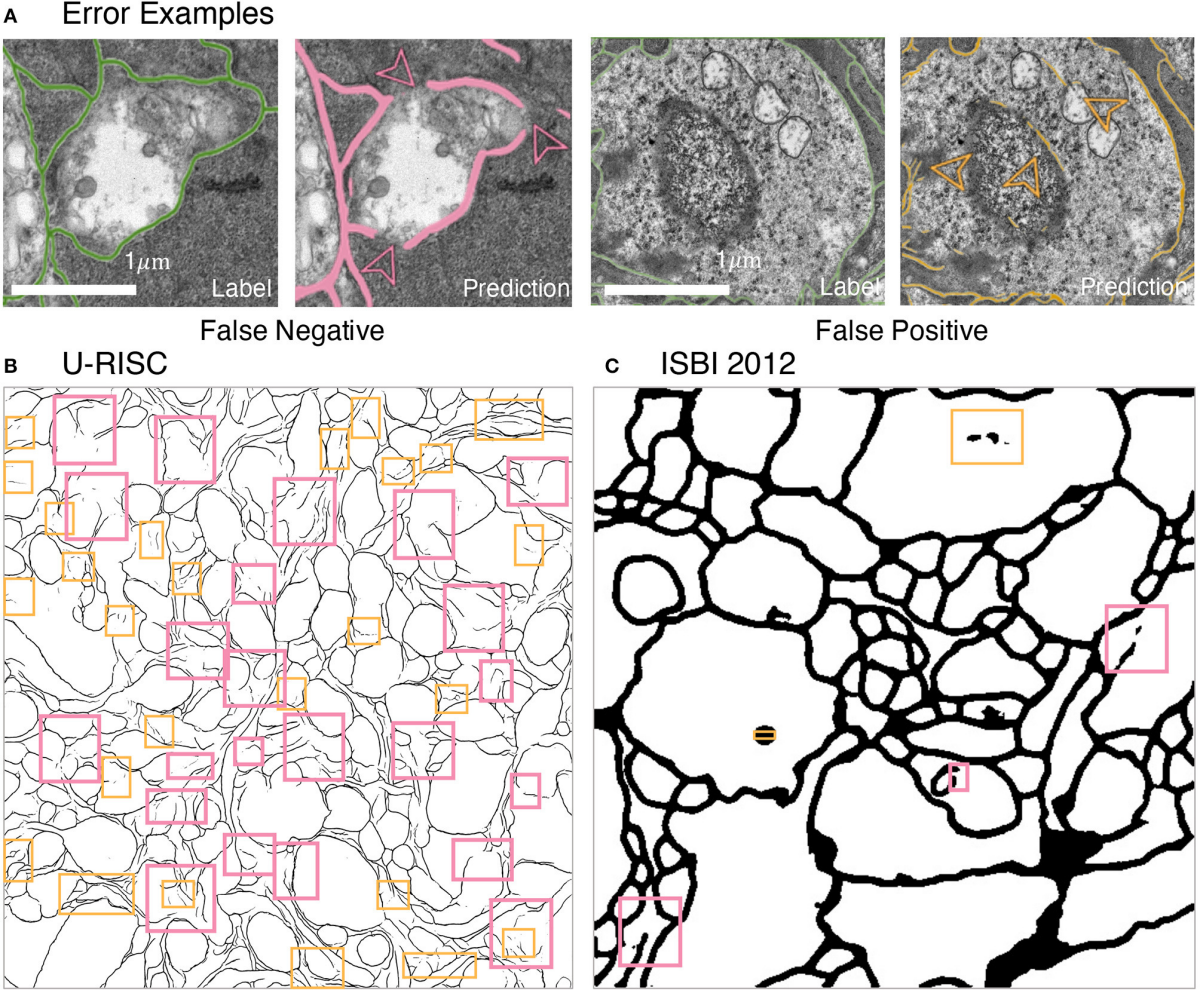
Errors in segmentation predictions of U-RISC and ISBI 2012. **(A)** The examples of false-positive and false-negative errors. **(B,C)** The examples of two errors in the segmentations of U-RISC and ISBI 2012. Pink arrows and lines represent false-negative errors, and orange represents false-positive errors.

What are the unique challenges brought by U-RISC to deep learning algorithms? Two types of errors were analyzed first: false-positive errors, which led to incorrect membrane predictions, and false-negative errors, which caused incontinuity in the cell membrane. According to our analysis, both false-positive errors (pink boxes) and false-negative errors (orange boxes) were common in the U-RISC, which were rare in ISBI 2012 (Figures 6B,C). More examples can be found in Figure 6 and Supplementary Figures 1–3. Further investigations for the networks are required to explore the reason and find ways to reduce the errors.

### Attribution Analysis of the Deep Learning Method on U-RISC and ISBI 2012

**Figure 7:**
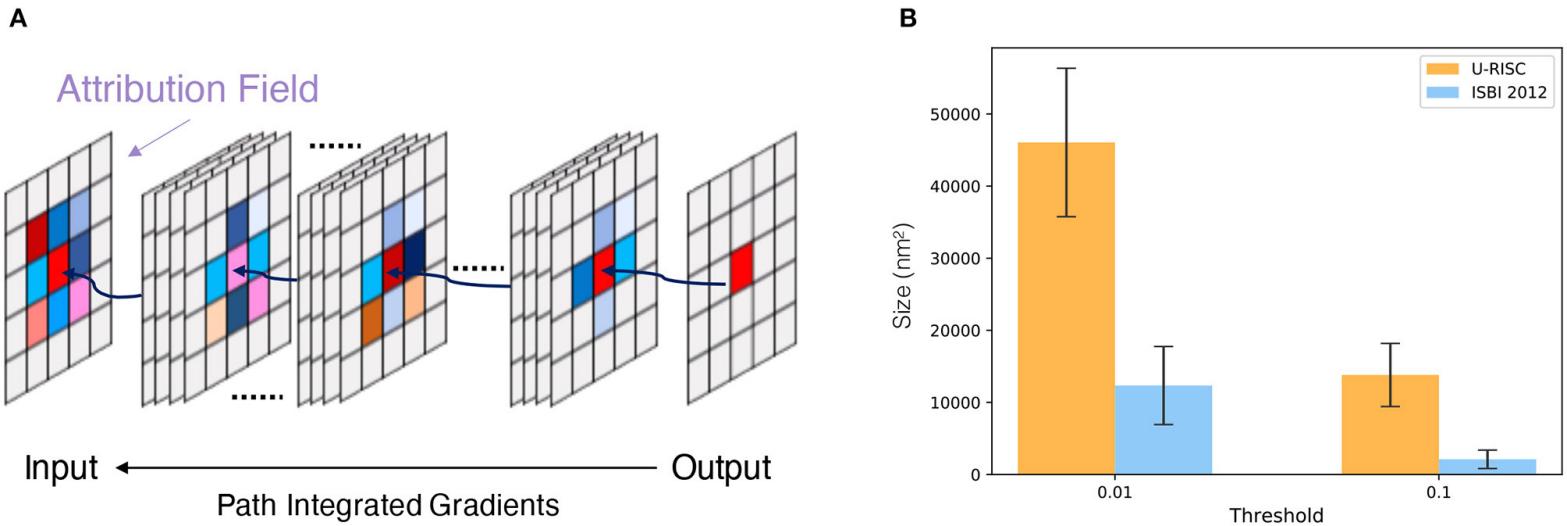
Attribution analysis. **(A)** Integrated gradients (IG) attribution method. **(B)** Statistics of attribution filed for U-RISC and ISBI 2012.

To acquire a deeper understanding of the different performances in U-RISC and ISBI 2012, we performed an attribution analysis (Ancona et al., 2019) on the trained U-Net. We selected the gradient-based attribution method, the IG (Sundararajan et al., 2017), which is widely applied to explainable artificial intelligence, such as understanding feature importance (Adadi and Berrada, 2018), identifying data skew (Clark et al., 2019), and debugging model performance (Guidotti et al., 2018). In brief, IG aims to explain the relationship between predictions and input features based on gradients (Figure 7A). The IG output is plotted in Attribution Fields to reflect their contribution to the final prediction. In the heatmap, each pixel was assigned with a normalized value between [−1, 1]. With IG, we analyzed the attribution field of each predicted pixel of U-Net in U-RISC and ISBI 2012. Color and shade were used to represent the normalized contribution values in attribution fields (Figure 7B). For a fair comparison between U-RISC and ISBI 2012, areas of pixel attribution fields, *S*_*k*_ were converted to physical size according to their respective resolutions.

**Figure 8:**
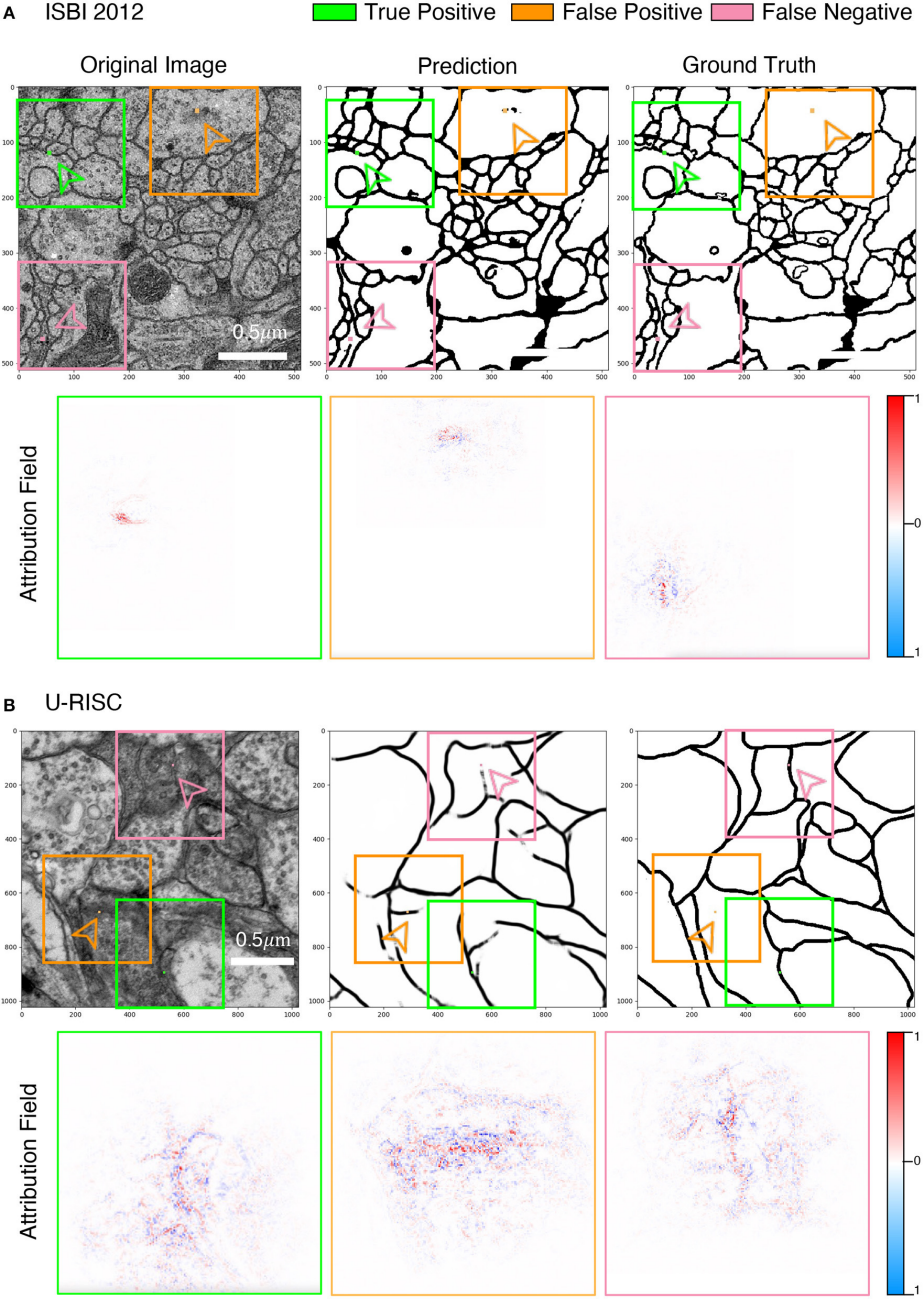
Attribution analysis. **(A,B)** Attribution fields of ISBI 2012 and U-RISC dataset. The first line represents the original image, network prediction result, and annotation respectively. The pixels pointed by green (correct cell membrane pixel), orange (false-positive predicted pixel), and pink (false-negative predicted pixel) arrows are the prediction points used in the attribution method. Images in the three-color boxes with the same size in the second line represent the attribution field corresponding to the above three pixels. Blue indicates that the network is likely to predict the pixels as the cell membrane, while the opposite is indicated by red.

Figure 8 shows the examples of attribution fields, where bounding boxes with different colors represented different pixel classifications, green for a correct predicted pixel, orange for a false-positive error, and pink for a false-negative error. More examples can be found in Supplementary Figures 4–6. We noticed that the areas of attribution fields *S*_*k*_ of two datasets were both relatively minor to the whole images (Figure 7B). For example, at the threshold of *k* > 0.01, the *S*_*k*_ of the correct cases accounted for only 5.1 and 0.8% relative to the whole image (the green bounding boxes in Figure 8). This suggested that the U-Net would focus on local characteristics within small areas of the images when making predictions. In addition, we found that the averaged *S*_*k*_ of each predicted pixel in U-RISC was significantly larger than that in ISBI 2012, specifically 46,000 *n*m^2^ in U-RISC and 10,300 *n*m^2^ in ISBI 2012. Taken together, the U-Net would predict cell membrane according to local information around the pixel, and the average attribution field was larger in U-RISC than that in ISBI 2012. All of these indicate that more information is required for the segmentation in U-RISC.

### U-Net-Transfer Model Achieves the SOTA Result on the U-RISC Benchmark

**Figure 9:**
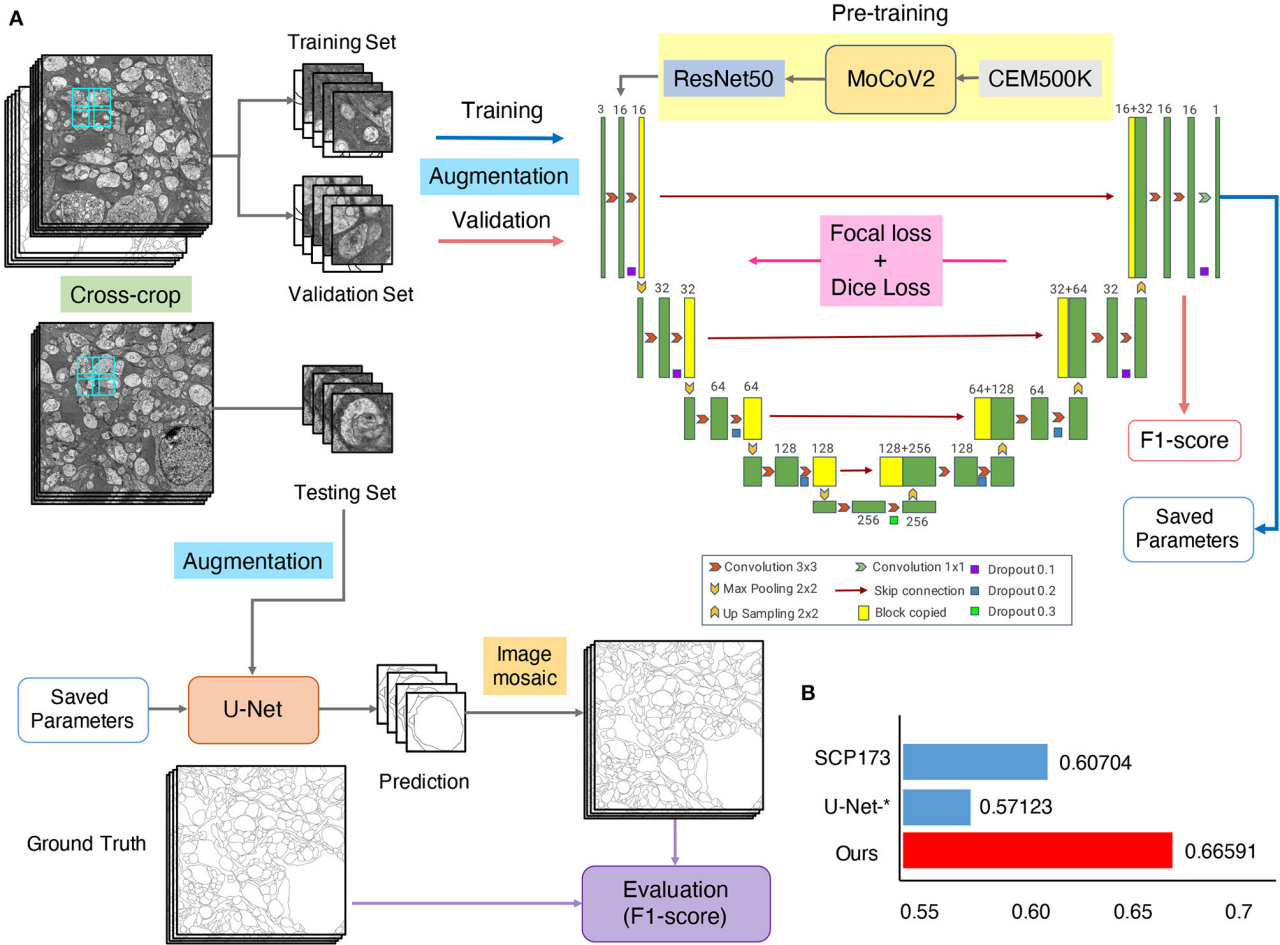
The U-Net-transfer method achieves the best performance on U-RISC. **(A)** The pretraining, training, and testing processing for U-Net. **(B)** The comparison of the F1-scores. “SCP-173” represents the top performance in the competition. U-Net-^*^ represents the performance in Table 2. This section represents the performance of the U-Net-transfer method.

Considering both the comprehensive analyses of competition and attribution analysis, we integrated outstanding methods to develop our method (Figure 9A). For basic segmentation architecture, we chose the U-Net due to its better characteristic extraction ability. Many valuable techniques were also considered, including a cross-crop strategy for saving computational resources and data augmentation to increase data diversity. We chose both focal loss and dice loss to deal with the imbalance of samples for the loss function design. Some parameters used for training were also optimized, such as batch-size/GPU (4) and the number of GPUs (8). For more details, please refer to Segmentation networks in Methods and materials. Especially, a recent study has shown that transfer learning with domain-specific annotated datasets could be effective in elevating deep learning models' performance (Conrad and Narayan, 2021). Therefore, we introduced a pretrained model, trained with MoCoV2 (Arar et al., 2020) on CEM500K (Conrad and Narayan, 2021). The segmentation result showed that the F1-scores of our method were 10% higher than the leader of the competition (0.66 vs. 0.61 in Table 2 and Figure 9B). Thus, we provide a new benchmark on the cellular membrane segmentation of U-RISC.

## Discussion

This article first proposed the U-RISC, a cell membrane EM dataset created through intensive and elaborate annotation. The dataset is characterized by the highest resolution and the largest single image size compared to the other current publicly available annotated EM datasets. Next, we organized a segmentation competition on U-RISC and proposed the benchmark. During the competition, we noticed that the performances of popular deep learning methods were far below that of humans, which motivated us to explore the causes. Thus, we carried out a comprehensive survey of the participants in the deep learning methods applied in the competition. To our surprise, methods, such as U-Net, LinkNet, and CASENet exhibited a significant drop of F1-score on the U-RISC compared to ISBI 2012, from 0.9 to 0.6. To explore the mechanisms underlying this discrepant performance, we introduced a gradient-based attribution method, the IG. Through attribution analysis of U-Net, we found that the average pixel attribution field of U-RISC is larger than that of ISBI, corresponding to the size of cellular structure, and both of them are relatively small to the whole image size. By integrating currently available methods, we improve the benchmark to 0.67, about 10% higher than the top leader from the competition. Based on the analyses in this article, here, we raise some considerations in the challenges for deep learning-based segmentation algorithms brought by U-RISC and propose several suggestions for improving the EM segmentation methods.

### Challenges for Deep Learning-Based Segmentation

Benchmark showed that the segmentation performance of deep learning algorithms on U-RISC was still far behind the human level. The U-RISC poses challenges for deep learning-based segmentation in the following aspects: (1) high computational costs needed to deal with large images, (2) the extreme sample imbalance caused by the low ratio of cellular membrane pixels in the whole image, and (3) side effects of typical data processing methods.

Deep learning itself is already a computationally intensive method. It would require more computational resources to process the images with a much larger size in the U-RISC. In practical terms, taking U-Net as an example, processing a 1,024 × 1,024 pixel image requires a GPU with 12GB memory. This memory is enough to deal with the images in ISBI 2012, of which the size is 512 × 512 pixels. But the size of a single image in the U-RISC is 9,958 × 9,959 pixels, which is far beyond the processing ability of the commonly used 12 GB memory GPU. Therefore, the additional computational burden brought by the U-RISC raises the first challenge for deep learning-based segmentation.

The problem of imbalanced samples widely exists in computational vision tasks (Li et al., 2010; Alejo et al., 2016; Zhang et al., 2020), which should be considered when designing algorithms. Cellular membrane segmentation is a typical situation of sample imbalance because the cellular membrane only occupies a small proportion of the whole cell structure. According to statistics, the pixels belonging to the cellular membrane account for 21.65% of the entire pixels of ISBI 2012. While the proportion in U-RISC is much smaller, 5.10%, making the U-RISC an extremely imbalanced dataset. Preexisting solutions were mainly proposed from several aspects: loss function design (Lin et al., 2017; Cui et al., 2019), data augmentation (Yoo et al., 2020), under/over-sampling (Fernández et al., 2018), and semantically multi-modal approaches (Zhu et al., 2020). However, even though the participants in the competition already used these approaches, the final results showed a limited improvement in segmentation. So, the imbalanced problem of U-RISC is yet to be solved and becomes another challenge for deep learning-based segmentation.

Proper data processing is essential and helpful for deep-learning algorithms. For example, a downsampling process on raw images with an enormous size is commonly adopted in the segmentation tasks (Thakkinstian et al., 2005; Chen et al., 2014). In Track 2 of our competition, we used the downsampled dataset to reduce the computational consumption, as usual. Surprisingly, we found that the F1-score of the same method dropped and the overall performance was also decreased in Track 2 compared to Track 1. We speculated that the key reason might be the degradation of image quality from Track 1 to Track 2. We confirmed the quality reduction through four representative indices, including Brenner (Subbarao and Tyan, 1998), SMD2 (Thakkinstian et al., 2005), Variance (Saltelli et al., 2010), and Vollath (2008) (shown in Appendix Figure). More cautions should be paid when using traditional data processing methods, and more advanced data processing theories are expected from this point of view.

### Suggestions for the Improvement of Segmentation Methods

To some degree, increasing computational resources are possible ways to cope with the challenges mentioned above. However, it might not be easy for all the community researchers to access sufficient computational power; therefore, innovations in algorithms are still crucial for our future success. To improve the performance of deep learning in EM segmentation, we provide several suggestions for developing deep-learning algorithms from the following perspectives: model design, training techniques, data processing, loss function design, and visualization tools.

#### Model Design

As shown in the attribution analysis, the current models for segmentation, such as U-Net (Ronneberger et al., 2015), Efficientnet (Tan and Le, 2019), and CASENet (Yu et al., 2017), are designed to focus on the local information to make predictions. However, in a high-resolution image, other structures, like organelle membrane and synaptic vesicles, might share similar features with the cellular membrane on a local scale, which leads to false-positive results. Additionally, this constitutes one of the major error types in the competition. Therefore, it might not be enough for the classifiers of a model to make correct decisions with only local features. Multi-scale features can increase the learning ability of the neural network, and studies have shown that models using global information could improve the performance greatly (Liu et al., 2018, 2020; Chen et al., 2019). Therefore, more global information could also be considered in the future design of the segmentation network.

#### Training Techniques

Skillful training techniques can also be helpful in improving segmentation performance. According to our survey, a two-stage training strategy could be much better than a single-stage training strategy. A recent study also suggests that pretraining with domain-specific datasets can help network learning domain features (Conrad and Narayan, 2021). Besides that, much experience can be learned from the existing training methods. The Hypercolumns module (Hariharan et al., 2015) is used to accelerate the convergence of training by combining features at different scales, and the combination of features from different scales can help bring in global information. The ScSE (Roy et al., 2018) module introduces an attention mechanism into the network, thus, bringing in global information. Hybrid architectures can also be considered because of their ability to expand the receptive field (Goceri, 2019). In a word, improvement can be made at the phase of the training by utilizing advanced training techniques.

#### Data Processing

Data processing is commonly used in deep learning, while traditional downsampling methods were shown to have side effects in the competition. To alleviate the side effects, some quality enhancing methods for downsampled images could be expected, such as edge and region-based image interpolation algorithms (Hwang and Lee, 2004; Asuni and Giachetti, 2008), low bit rate-based approaches (Lin and Dong, 2006; Wu et al., 2019), and quality assessment research (Wang et al., 2003; Wang and Bovik, 2006; Vu et al., 2018). Meanwhile, other data processing methods can also be taken into account. For example, in data augmentation, by augmenting the training data randomly (such as multi-scale and multi-angle), the dependence of the model on specific attributes can be reduced, which can be beneficial in EM segmentation with many imbalanced samples.

#### Loss Function Design

Loss function design is another important part of deep learning. But many current loss functions have their own disadvantages in our competition. For example, dice loss (Dice, 1945) was designed to optimize F1-score directly, without consideration of data imbalance. Focal loss (Lin et al., 2017) and BCE loss (Cui et al., 2019) were used in the competition to care more about data imbalance by giving different penalties according to sample difficulty, but the improvement was limited as shown by the results. A better design of loss function should take an overall consideration of both the sample imbalance and evaluation criteria. Most of the common evaluation criteria, such as the F1-score, a pixel-based statistic, are inconsistent with the human subjective feeling to some extent. It might be a major cause of the performance gap between humans and algorithms. Some other structure-based criteria have appeared, such as V-Rand and V-info (Arganda-Carreras et al., 2015) that integrate skeleton information of cell membrane and ASSD (Heimann et al., 2009), considering the distance of point sets.

#### Visualization Tools

Visualization tools can help us have a better understanding of the network. In this article, from IG, we could learn the attribution fields of U-Net from the view of gradient, which inspires us to improve deep learning methods by paying more attention to global information. In comparison, many other visualization tools start from other characteristics of the network. Layer-wise relevance propagation (LRP) (Bach et al., 2015) and deep Taylor decomposition (DTD) (Montavon et al., 2017) get attribution distribution by modifying the propagation rules. The information-based method, the IBA (Schulz et al., 2020) restricts the flow of information to accomplish attribution fields. Combining different visualization tools can help promote much more insightful inspiration in improving deep learning methods.

Overall, we provide an annotated EM cellular membrane dataset, U-RISC, and its benchmark. This indeed brings many challenges in deep learning and promotes the development of deep learning methods for segmentation.

## Data Availability Statement

The datasets presented in this study can be found in online repositories. The names of the repository/repositories and accession number(s) can be found below: https://github.com/EmmaSRH/U-RISC-Data-Code.

## Author Contributions

RS, WW, KD, and TJ contributed to conception and design of the study. RS, WW, LH, and ZL organized the database. All authors contributed to manuscript revision, read, and approved the submitted version.

## Funding

This work was partially supported by the Natural Science Foundation of China under contract 62088102.

## Conflict of Interest

The authors declare that the research was conducted in the absence of any commercial or financial relationships that could be construed as a potential conflict of interest.

## Publisher's Note

All claims expressed in this article are solely those of the authors and do not necessarily represent those of their affiliated organizations, or those of the publisher, the editors and the reviewers. Any product that may be evaluated in this article, or claim that may be made by its manufacturer, is not guaranteed or endorsed by the publisher.

## Footnotes

^1^ https://www.biendata.xyz/competition/urisc/

## Supplementary Material

Supplementary Figure 1 Supplements for segmentation predictions of U-RISC.

Supplementary Figure 2 Supplements for segmentation predictions of ISBI 2012.

Supplementary Figure 3 Supplements for segmentation predictions of U-RISC (Track 2).

Supplementary Figure 4 Supplements for attribution analysis on ISBI 2012.

Supplementary Figure 5 Supplements for attribution analysis on U-RISC.

Supplementary Figure 6 Supplements for attribution analysis on U-RISC (Track 2).

Appendix Figure Differences between the original image and downsampled image. **(A)** The crop of the original image. **(B)** The crop of downsampled images at the same position. The gray-scale histograms are calculated on A and B. **(C)** The scores of definition indices calculated on the whole U-RISC dataset before and after downsampling. Details of indices are described in Methods and materials.
