# Supplementary figures and images for: U-RISC: An Annotated Ultra-High-Resolution Electron Microscopy Dataset Challenging the Existing Deep Learning Algorithms
Source: Front Comput Neurosci. 2022 Apr 11;16:842760. doi: 10.3389/fncom.2022.842760 (PMC9038176; doi:10.3389/fncom.2022.842760)

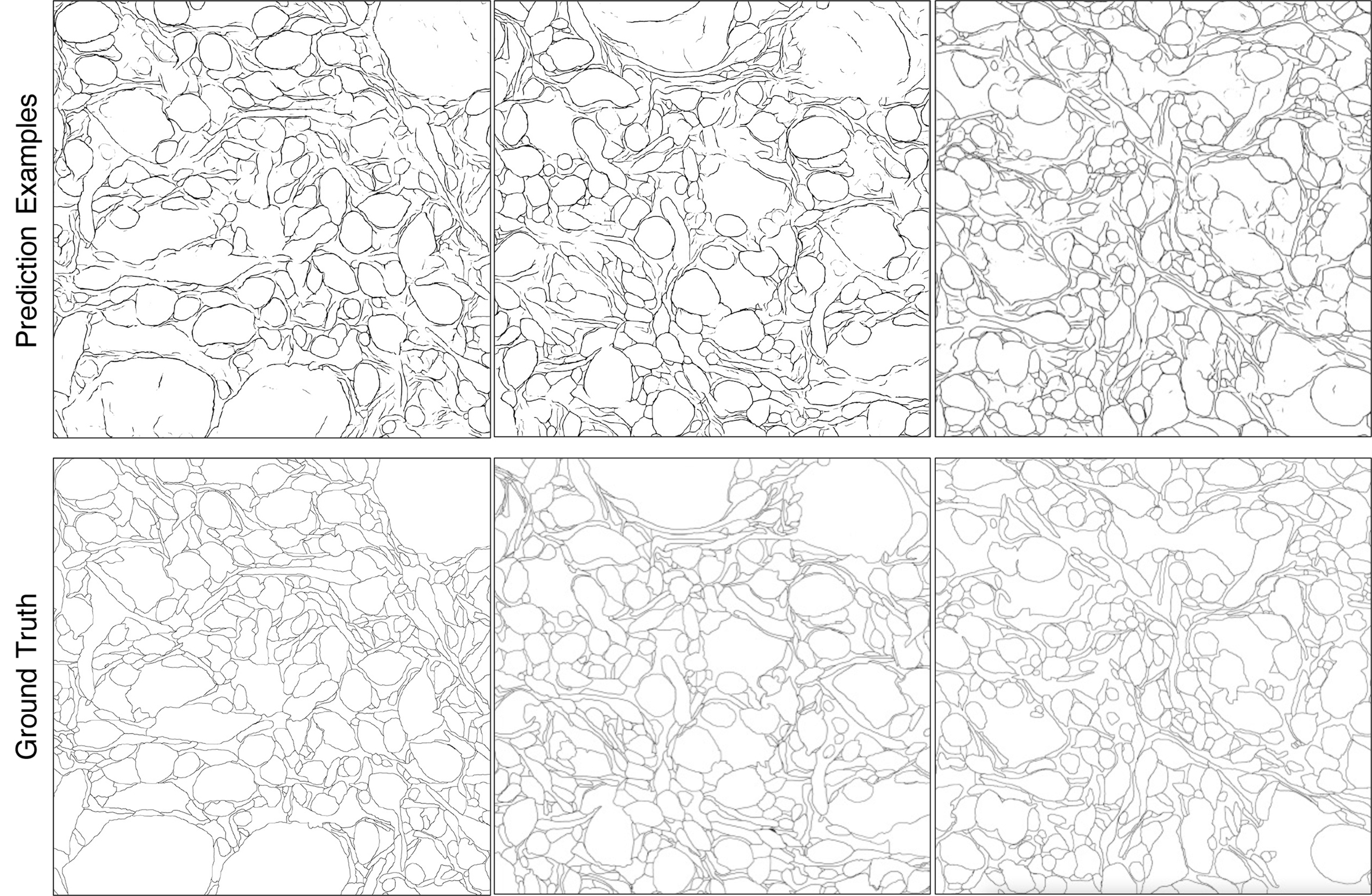

Supplement: Supplementary Figure 1 — Supplements for segmentation predictions of U-RISC. [file Image_1.jpg]

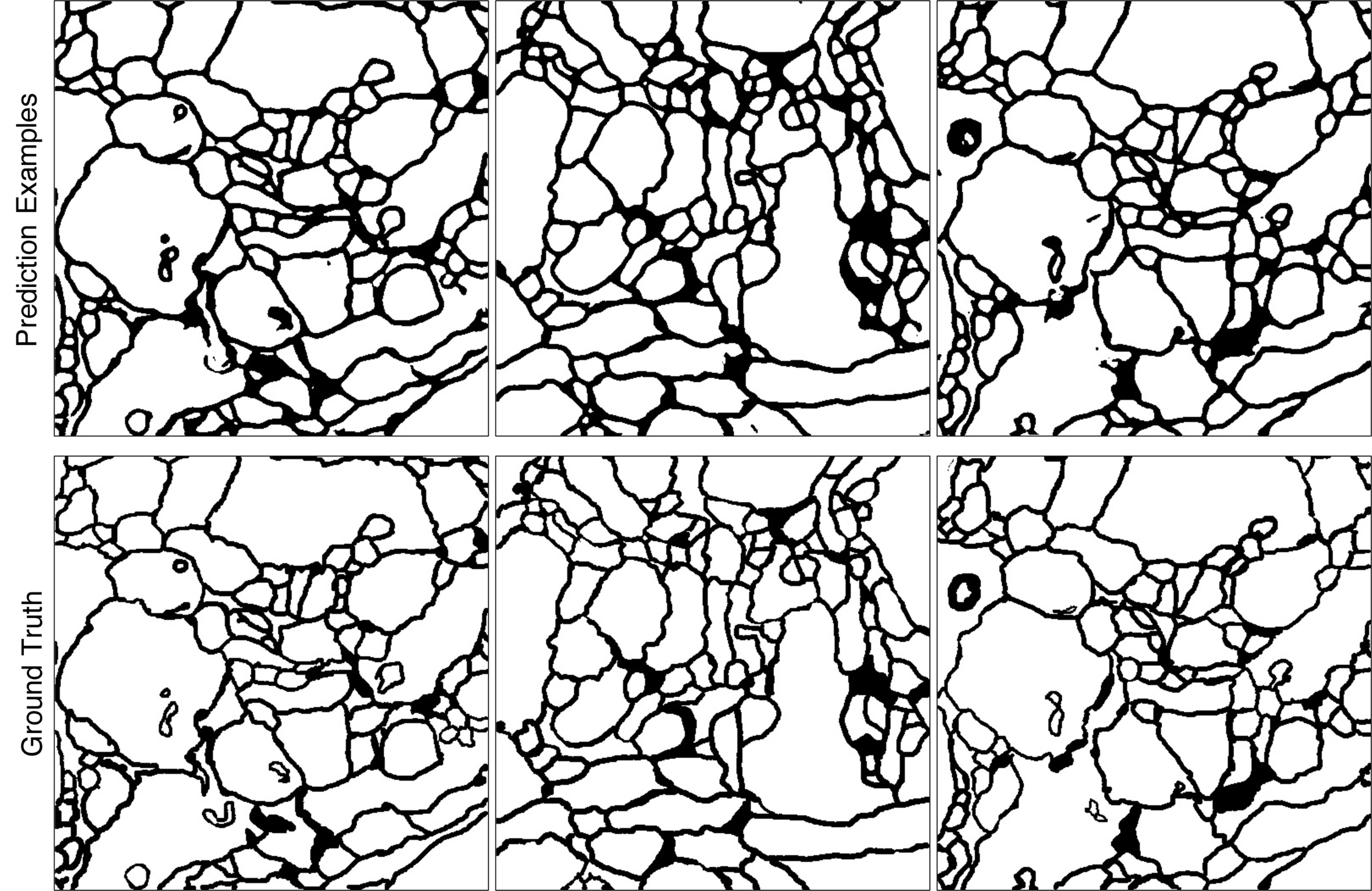

Supplement: Supplementary Figure 2 — Supplements for segmentation predictions of ISBI 2012. [file Image_2.jpg]

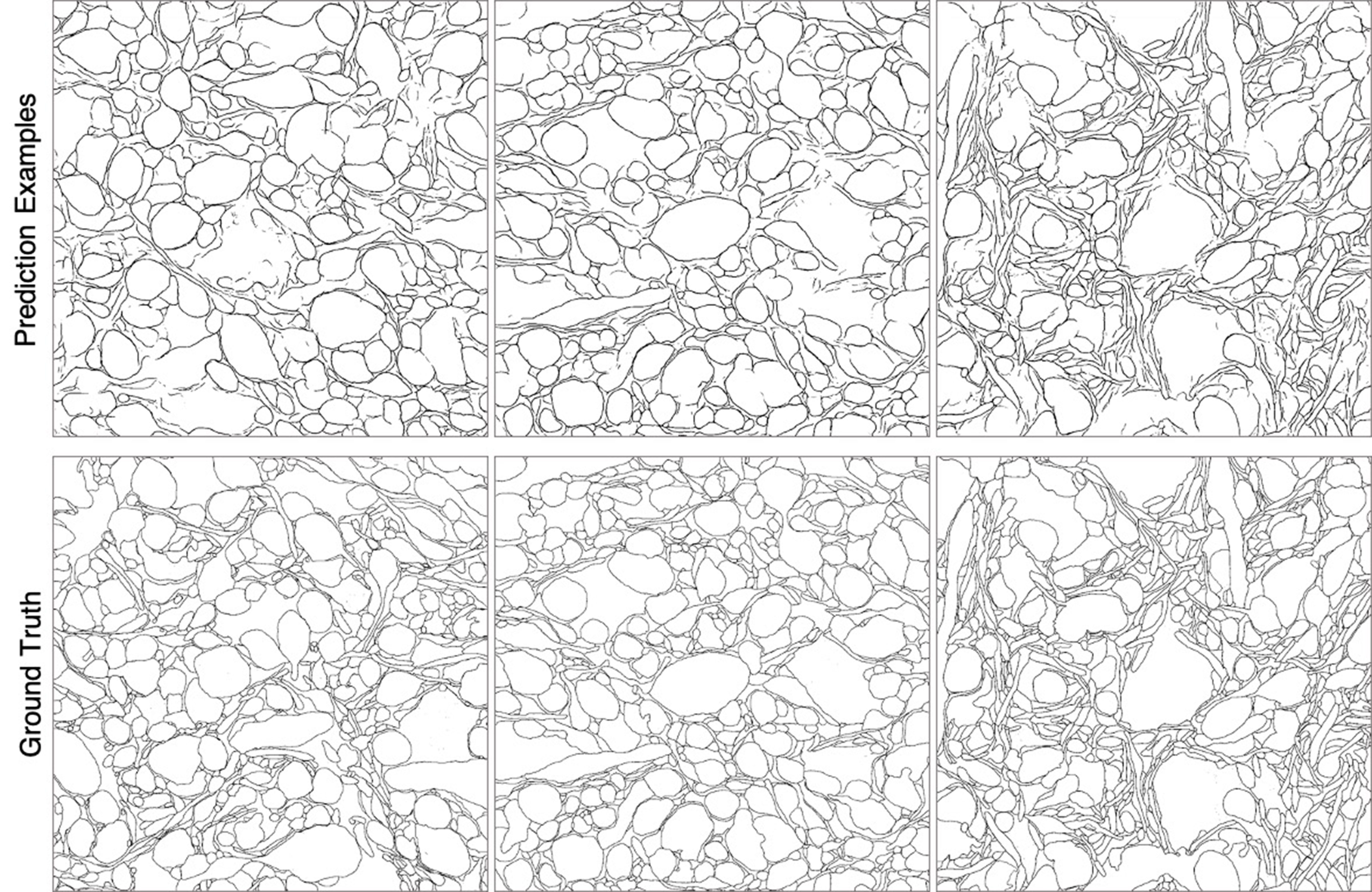

Supplement: Supplementary Figure 3 — Supplements for segmentation predictions of U-RISC (Track 2). [file Image_3.jpg]

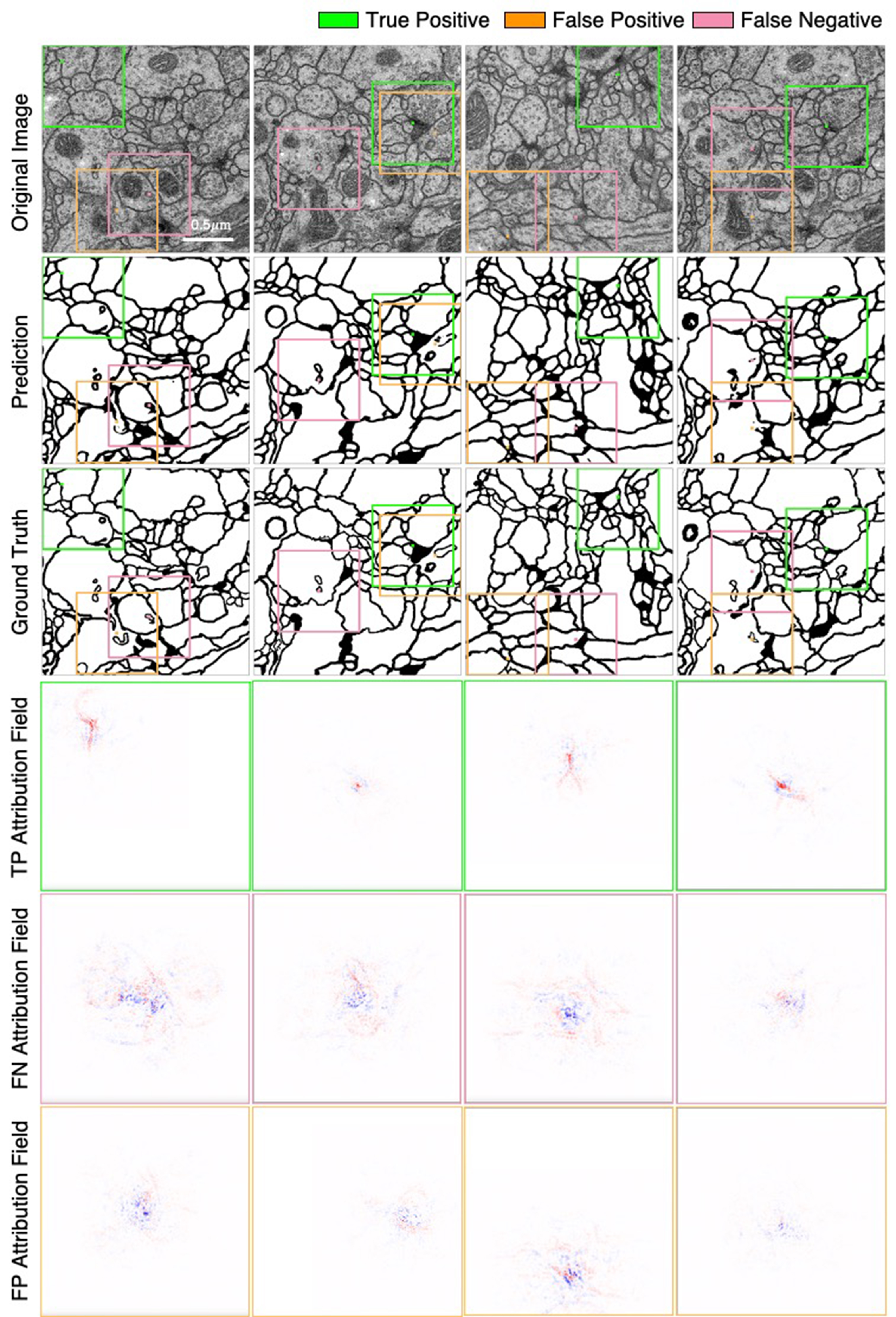

Supplement: Supplementary Figure 4 — Supplements for attribution analysis on ISBI 2012. [file Image_4.jpg]

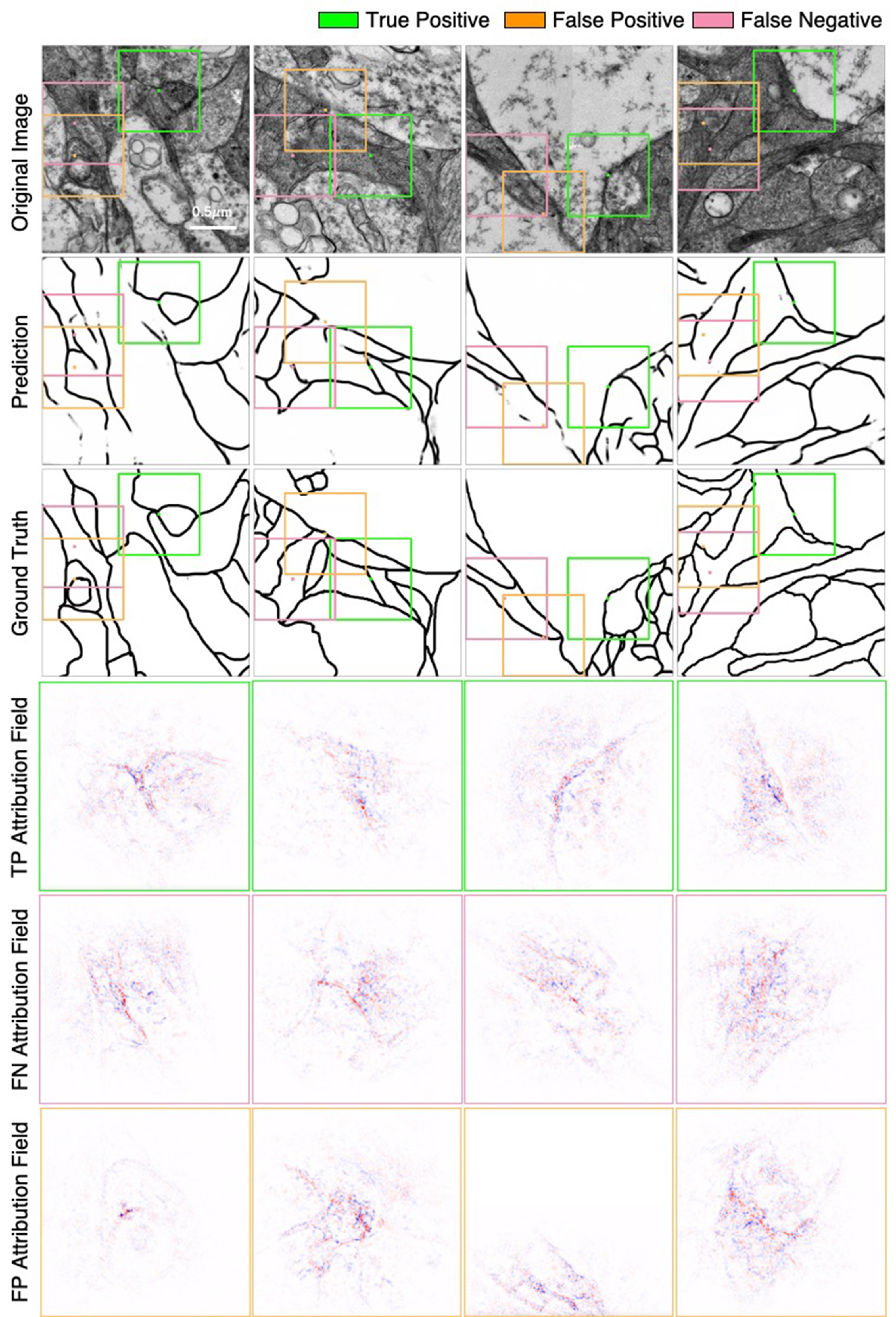

Supplement: Supplementary Figure 5 — Supplements for attribution analysis on U-RISC. [file Image_5.jpg]

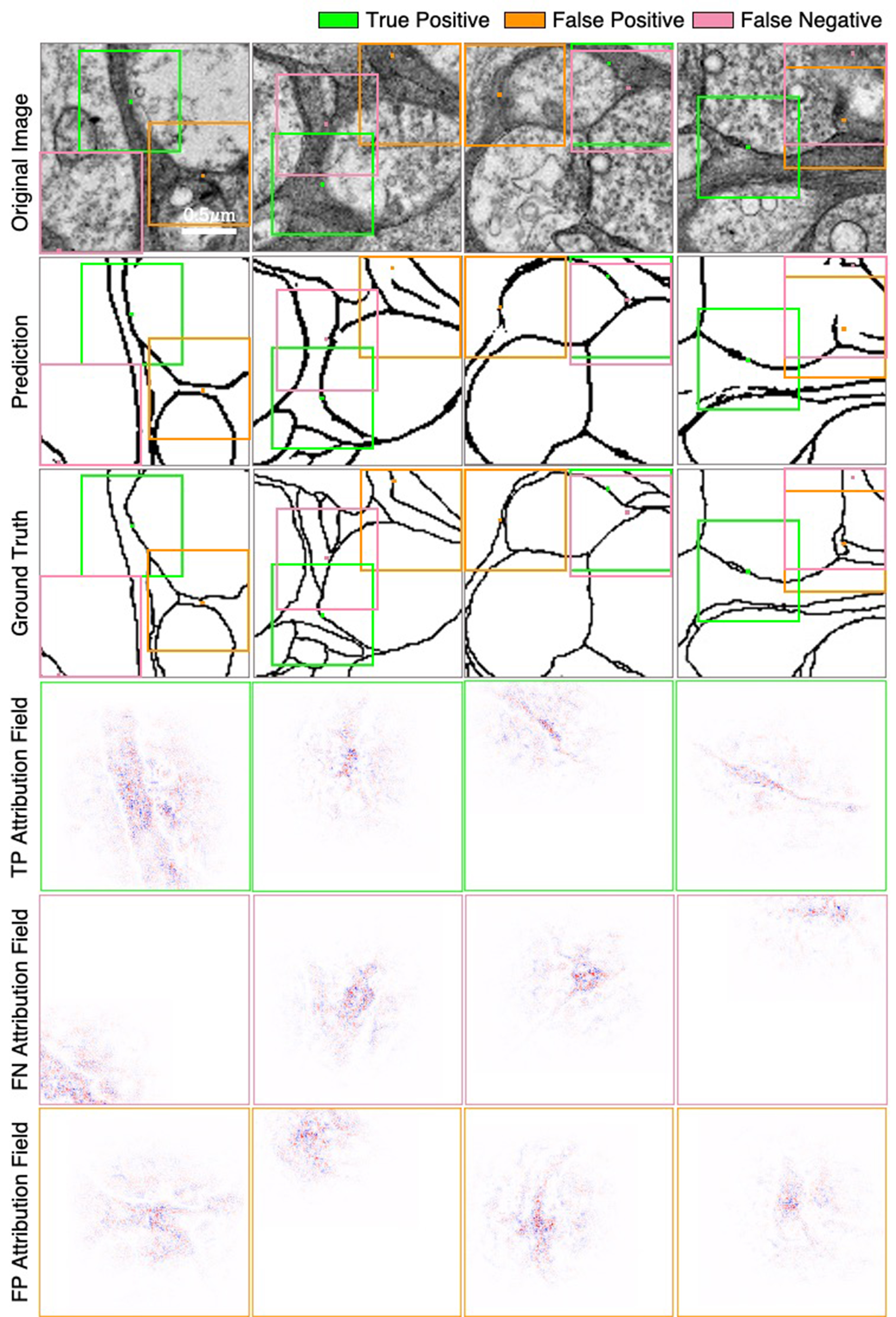

Supplement: Supplementary Figure 6 — Supplements for attribution analysis on U-RISC (Track 2). [file Image_6.jpg]
