# Supplementary material for: U-RISC: An Annotated Ultra-High-Resolution Electron Microscopy Dataset Challenging the Existing Deep Learning Algorithms
Source: Front Comput Neurosci. 2022 Apr 11;16:842760. doi: 10.3389/fncom.2022.842760 (PMC9038176; doi:10.3389/fncom.2022.842760)
Supplement: Appendix Figure — Differences between the original image and downsampled image. (A) The crop of the original image. (B) The crop of downsampled images at the same position. The gray-scale histograms are calculated on A and B. (C) The scores of definition indices calculated on the whole U-RISC dataset before and after downsampling. Details of indices are described in Methods and materials. [file Data_Sheet_1.pdf]

The Effects of Downsampling on U-RISC Dataset

To our best knowledge, downsampling is an effective approach to process images with enormous size in segmentation tasks. With the purpose of investigating the effects of downsampling on image definition quality, we analyzed the gray-scale histograms on a group of original and downsampled images in U-RISC and then calculated the definition values of images in Track 2.

We first found that downsampling produced numerous sharp changes between adjacent gray values (Appendix Figure C). In our analysis, the mutations might engender the texture information in the image to be blurred. For example, the bilayer structure of the membrane disappeared after downsampled (Appendix Figure A,B), and some of the cell membranes which were hard to recognized became obscured. We then found that the defining quality of the images in Track 2 became lower after downsampling. Four definition criteria (see Materials and Methods) of all the images in Track 2 were calculated. Brenner, SMD2, Variance, and Vollath are common indexes to show the gray value change between adjacent pixels. Results suggested that image indexes were significantly decreased after downsampling (Appendix Figure D), and all the four indexes dropped about ten times. Therefore, the analysis indicated that images were heavily blurred after the downsampling operation. However, downsampling preserved more global shape of membranes which is also an important information for membrane segmentation (Supplementary Figure 3). Therefore, although the F1 score in Track2 dropped due to the loss of details, the segmentation results accord more with human's intuition. We also analyzed the attribution field of Tarck2 (Supplementary Figure 6). Compared with Track1, results of Track2 show larger size of pixel attribution and much lower computational cost.

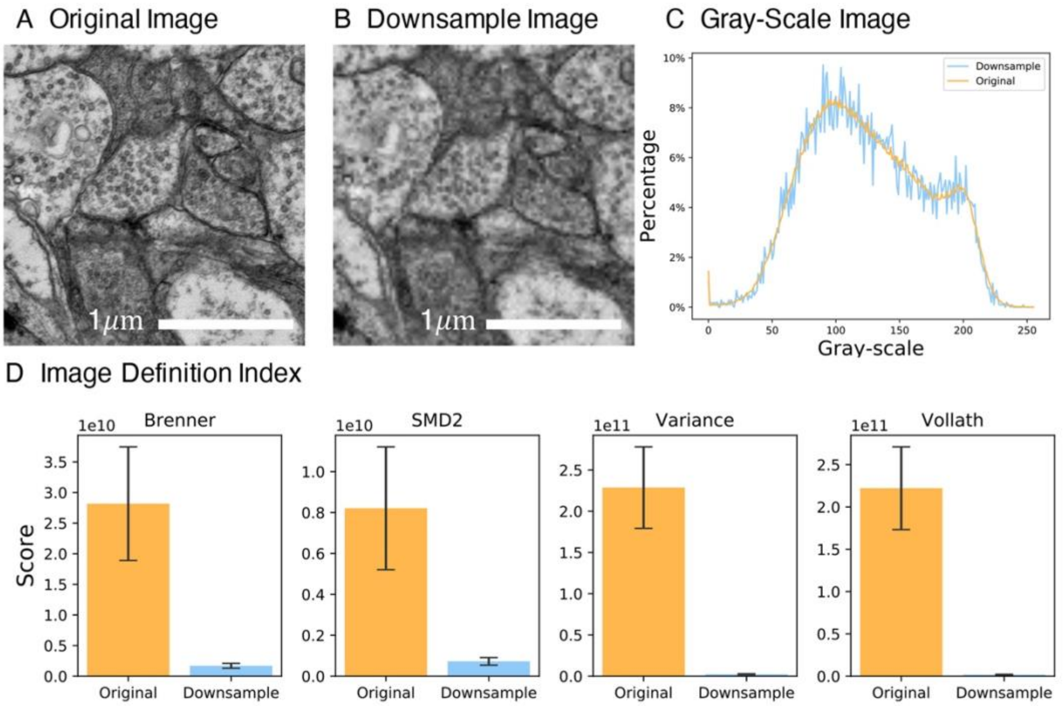

Appendix Figure. Differences between the original image and downsampled image. **(A)** The crop of the original image. **(B)** The crop of downsampled images at the same position. The gray-scale histograms are calculated on A and B. **(C)** The scores of definition indices calculated on the whole U-RISC dataset before and after downsampling. Details of indices are described in Methods and materials.
